# The regulation of PSI cyclic electron transport by both plastoquinone and ferredoxin redox states: correlation with the rate of proton motive force utilization

**DOI:** 10.3389/fpls.2025.1626163

**Published:** 2025-08-22

**Authors:** Hayato Satoh, Yuri Ohara, Guy Hanke, Kentaro Ifuku, Ginga Shimakawa, Yuji Suzuki, Amane Makino, Kenichi Morigaki, Chikahiro Miyake

**Affiliations:** ^1^ Graduate School of Agricultural Science, Kobe University, Rokkodai, Nada-Ku, Kobe, Japan; ^2^ School of Biochemistry and Chemistry, Queen Mary University of London, London, United Kingdom; ^3^ Graduate School of Agriculture, Kyoto University, Kitashirakawa Oiwake-cho, Sakyo-ku, Kyoto, Japan; ^4^ Faculty of Agriculture, Iwate University, Morioka, Ueda, Morioka, Japan; ^5^ Graduate School of Agricultural Science, Tohoku University, Sendai, Japan; ^6^ Institute for Excellence in Higher Education, Tohoku University, Sendai, Japan

**Keywords:** photosyntheis, photosynthetic electron transport, cyclic electron transport around PSI, electrochromic shift, plant nutricion, nitrogen

## Abstract

The capacity of plants to protect themselves from stress and efficiently assimilate CO_2_ depends on dynamic regulation of photosynthetic electron transport pathways. In the cyclic electron transport around photosystem I (PSI-CET), the ferredoxin (Fd) reduced by PSI donates electrons to plastoquinone (PQ), which then enter the pathway of photosynthetic linear electron transport (LET). It has been postulated that PSI-CET generates the additional proton motive force needed to drive sufficient ATP synthase activity for CO_2_ assimilation. The rate of PSI-CET relative to LET responds dynamically to environmental conditions and the metabolic demands of the chloroplast, but the mechanism for this regulation is still under debate. The rate of PSI-CET has been quantified as the oxidation rate of reduced Fd that exceeds the oxidation rate due to LET, which we term vFd(CET). In this study, the effects of the redox states of both PQ and Fd on vFd(CET) were analyzed in relation to the dependence of CO_2_ assimilation on light intensity in the C3 plant *Helianthus annuus*. In contrast to the rate of CO_2_ assimilation, the rate of PSI-CET demonstrated phases of acceleration and deceleration as the light intensity increases. The acceleration of vFd(CET) correlated with reduction state of Fd, while the deceleration correlated with reduction state of PQ. Plants grown with high nitrogen exhibited higher CO_2_ assimilation rates, more oxidized PQ and greater vFd(CET). Furthermore, a strong correlation was observed between vFd(CET) and the usage rate of proton motive force. These findings demonstrate that *in vivo*, vFd(CET) is regulated by the redox states of both Fd and PQ.

## Introduction

Oxygenic photosynthesis can be divided into two main reactions: the light reaction, which synthesizes ATP and NADPH, and the dark reaction, which uses these molecules to assimilate CO_2_. The light-driven electron transport reaction occurs when light energy is absorbed by two photosystems, PSI and PSII, which are located in the thylakoid membrane of chloroplasts. In PSII, the absorbed light energy excites the reaction center Chl P680, thereby initiating the oxidation of water through the Mn cluster. The electrons produced from water oxidation are transported through plastoquinone (PQ), the cytochrome (Cyt) *b*_6_/*f* complex, plastocyanin (PC), and PSI to ferredoxin (Fd). Subsequently, Fd-NADP oxidoreductase (FNR) reduces NADP^+^, forming NADPH, with Fd acting as the electron donor. This series of electron transfers, from water oxidation to NADPH production, is referred to as linear electron transport (LET). During LET, H^+^ are released into the thylakoid lumen during water oxidation in PSII and as electrons are transported from the reduced PQ to the Cyt *b*_6_/*f* complex and then to PC. Consequently, a proton gradient (ΔpH) and membrane potential (ΔΨ) are established between the thylakoid lumen and the stroma, both of which serve as the proton motive force (pmf) to drive ATP synthase (Cruz et al., 2004; Tikhonov and Vershubskii, 2014). In C3 plants, the NADPH and ATP produced by these light reactions power so called “dark reactions”, such as CO_2_ assimilation and photorespiration (Genty et al., 1990; Miyake, 2020).

In addition to LET, cyclic electron transport around PSI (PSI-CET) has been proposed to play a role in photosynthesis based on findings from *in vitro* studies (Arnon et al., 1954; Arnon, 1959; Tagawa et al., 1963). In PSI-CET, Fd, which is reduced by PSI, donates electrons back to oxidized PQ, with the reduced PQ then re-entering LET to contribute to pmf, thus supporting additional ATP synthesis (Cramer and Zhang, 2006). A number of mediators have been proposed as potential intermediates in PSI-CET, including FNR, the Cyt *b*_6_/*f* complex, NAD(P)H dehydrogenase (NDH), and PGR5/PGRL1 (Sazanov et al., 1998; Joliot and Joliot, 2002; Munekage et al., 2002; Kurisu et al., 2003; Laisk et al., 2010; Miyake, 2010; Strand et al., 2016; Fisher et al., 2019). However, their mechanistic roles and relative contributions remain to be confirmed.

In order to elucidate the role of the mediators in PSI-CET in C3 plant leaves, it is first necessary to establish the existence and characteristics of PSI-CET *in vivo* within intact leaves. This necessitates the presentation of evidence pertaining to electron transport that is independent of LET and in accordance with the theoretical model of PSI-CET (Allen, 2003). The electron transport rate of Fd (vFd), the stromal electron carrier in both LET and PSI-CET, was assessed *in vivo* in intact *Arabidopsis thaliana* leaves (Ohnishi et al., 2023; Maekawa et al., 2024). In anticipation of fluctuations in the PSI-CET rate, we examined changes in the relationship between the rate of LET and vFd in response to alterations in the intercellular partial pressure of CO_2_ (Ci) within the leaves. A plot of vFd against LET rate revealed a bimodal distribution, with vFd proportional to the rate of LET at low Ci levels. However, at higher Ci, the increase in vFd exceeds that due to LET, indicating an Fd oxidation reaction independent of LET (Ohnishi et al., 2023; Maekawa et al., 2024).

The correlation between increased vFd at high Ci, and a reduced PQ pool was also observed in *Arabidopsis* crr4 mutants, which lack the NDH component of PSI-CET. This indicates that the dominant Fd-oxidizing reactions in these conditions are independent of not only LET, but also NDH dependent PSI-CET.

The effects of Fd redox state on LET-independent and Fd-dependent electron transfer have also been investigated. The analysis of Ci dependence on LET demonstrated that reduced Fd levels remained stable even when Ci decreased, leading to decreased CO_2_ assimilation (Ohnishi et al., 2023; Maekawa et al., 2024). The suppression of the dark reaction as an electron sink results in a decrease in both luminal pH and PQ oxidation by the Cyt *b*_6_/*f* complex, which limits electron transport to PSI (Miyake, 2020; Wada et al., 2020). Moreover, the accumulation of reduced PQ inhibits the Cyt *b*_6_/*f* complex’s Q-cycle (through lack of oxidized PQ substrate), thereby suppressing the further oxidation of reduced PQ. This mechanism is referred to as “reduction-induced suppression of electron flow,” or RISE (Shaku et al., 2016; Shimakawa et al., 2018). This down-regulation of the Cyt *b*_6_/*f* complex limits the accumulation of electrons on the PSI acceptor side (Furutani et al., 2023; Shimakawa et al., 2024). This is the likely reason why the reduction state of Fd remains constant even in the lower Ci. A PGR5-deficient *Arabidopsis* mutant was utilized, which is unable to maintain ΔpH due to elevated proton conductance, resulting in a lower ΔpH compared to the wild type (DalCorso et al., 2008; Furutani et al., 2023). The PGR5-deficient mutant exhibited Fd in a more reduced state than the wild type, and LET-independent vFd was greater (Maekawa et al., 2024). It is noteworthy that this increase in vFd could only be measured in conditions of oxidized PQ, irrespective of Fd redox state. These findings corroborate the hypothesis that a rate of Fd-oxidation, independent of that driven by LET, adheres to the theoretical PSI-CET model, with the rate represented by the following equation: k × PQ × Fd^-^ (Allen, 2003). In this context, the rate constant k is contingent upon the catalytic efficiency and quantity of electron transport mediators from reduced Fd to oxidized PQ in PSI-CET. These observations serve to confirm the veracity of the PSI-CET hypothesis as an Fd-dependent electron transport process that is independent of LET in *Arabidopsis* leaves (Ohnishi et al., 2023; Maekawa et al., 2024).

The present study employed the C3 plant sunflower (*Helianthus annuus*) to evaluate this theoretical PSI-CET model and its physiological function. Sunflower leaves generally have a high nitrogen content in their leaves and exhibits a high CO_2_ assimilation capacity. Therefore, the dynamic range of changes in CO_2_ assimilation rate due to differences in leaf nitrogen content is large. This characteristic of sunflower makes it easier to observe changes in the redox state of electron carriers in the photosynthetic electron transport chain in response to variations in CO_2_ assimilation capacity. In our previously reported studies, the intercellular CO_2_ concentration (Ci) was decreased under constant light intensity (Ohnishi et al., 2023; Maekawa et al., 2024). In the wild-type *Arabidopsis thaliana*, the level of reduced ferredoxin (Fd) remained unchanged upon Ci reduction, while plastoquinone (PQ) became more reduced. As a result, the PSI-CET rate (vFd(CET)) decreased, indicating that oxidized PQ is required for the operation of PSI-CET (Ohnishi et al., 2023). Furthermore, the necessity of reduced Fd for PSI-CET activity is supported by the observation that the pgr5 mutant, which maintains a high level of reduced Fd, exhibits a higher vFd(CET) than the wild type (Maekawa et al., 2024). The redox states of PQ and Fd depend on the CO_2_ assimilation capacity of the intact leaf and respond dynamically to changes in light intensity. In the present study, we analyzed how changes in the redox states of PQ and Fd individually affect vFd(CET) and how they influence the functional activation of PSI-CET. To this end, we prepared two types of plants differing in leaf nitrogen content (control and High-N). Compared with control plants, High-N plants exhibited higher CO_2_ assimilation capacity, with more oxidized PQ and more reduced Fd under high light conditions. Moreover, increasing light intensity reduced both PQ and Fd, and the interaction of these reductions revealed their influence on vFd(CET). In addition, the rate of proton motive force (pmf) utilization for ATP synthesis (vH^+^) correlated with vFd(CET), supporting the role of PSI-CET in pmf formation as its physiological function.

## Materials and methods

### Plant materials

Sunflower (*Helianthus annuus*) plants were grown from seeds under standard air-equilibrated conditions with 30°C/25°C, light (16 h)/dark (8 h) cycles, 50 – 60% relative humidity, and 800 µmol photons m^-2^ s^-1^ light intensity in the control chamber, made from NK system (model: LPH-100LED-NCS). Seeds of sunflower for green manure purchased from TAKII & Co., Ltd (Kyoto, Japan). Seeds were sown in 0.8 dm^3^ pots containing a 1:1 mixture of vermiculite (Akagi Engei Co., Ltd, Gunma, Japan) and seeding-culture soil (Tanemakibaido, TAKII & Co., Ltd, Kyoto, Japan). The compost mainly contains N 380mg/L, P 290 mg/L, K 340mg/L. Plants were watered daily and fertilized (Hyponex 6-10-5; Hyponex Japan) only tap water for the control-grown plants and five times a week for the high-nitrogen (HN)-grown plants. All measurements were made using fully expanded leaf 3–4 weeks after sowing. All plants were adapted to the dark for > 30 min before measurements.

*Arabidopsis thaliana* wild type (Col-0) and the mutant, 35S;*PpFlv* no. 13 (Yamamoto et al., 2016) were grown from seeds under standard air-equilibrated conditions with 23°C/20°C, light (10 h)/dark (14 h) cycles, 50 – 60% relative humidity, and 70 - 85 µmol photons m^-2^ s^-1^ light intensity in control chamber.

### Biochemical assays

The contents of both chlorophyll (Chl) and nitrogen (N) in the leaves of sunflower plants were determined by the method reported in the previous study (Ohnishi et al., 2023). Chl was determined using Arnon’s method (Arnon, 1949). Nitrogen was determined using Kjeldahl methods. Rubisco was determined by formamide extraction of Coomassie Brilliant Blue R-250-stained bands corresponding to the large and small subunits of Rubisco separated by sodium dodecyl sulfate-polyacrylamide gel electrophoresis as described by Makino et al. (1985) and Suzuki et al. (2022). Contents of Chl, N, and Rubisco in the leaves of both the control- and HN-grown plants were shown in **Supplementary Table S1**.

### Simultaneous measurements of chlorophyll fluorescence, P700, plastocyanin, and ferredoxin with CO_2_/H_2_O-exchange

Chl fluorescence, P700, Plastocyanin, Ferredoxin, and CO_2_ exchange were simultaneously measured using Dual/KLAS-NIR (Heinz Walz GmbH, Effeltrich, Germany), and an infrared gas analyzer (IRGA) LI-6262/7000 (Li-COR, Lincoln, NE, USA) measuring system equipped with a 3010-DUAL gas exchange chamber at several atmospheric conditions (40 Pa CO_2_, 21 kPa O_2_) (Heinz Walz GmbH) was used (Ohnishi et al., 2023). The gases were saturated with water vapor at 14 ± 0.1°C. The leaf temperature was controlled at 25 ± 0.5°C (relative humidity: 50 – 60%). The light intensity at the upper position on the leaf in the chamber was adjusted to the indicated intensity. The net CO_2_ assimilation rate (A) and the dark respiration rate (Rd) were measured.

The Chl fluorescence parameters were calculated as follows (Baker, 2008): F_o_, minimum fluorescence from a dark-adapted leaf; F_m_, maximum fluorescence from a dark-adapted leaf; F_m_’, maximum fluorescence from a light-adapted leaf; Fs, fluorescence emission from a light-adapted leaf; the maximum quantum yield of PSII, Fv/Fm = (Fm – Fo)/Fm; the effective quantum yield of PSII, Y(II) = (F_m_’ - Fs)/F_m_’ (Genty et al., 1990); non-photochemical quenching, NPQ = (F_m_ - F_m_’)/F_m_’ (Bilger and Björkman, 1994); and PQ oxidized state derived from the lake model of Chl fluorescence (qL) = Y(II)/[1-Y(II)] × [(1 - Fv/Fm)/(Fv/Fm)] × (NPQ + 1) (Miyake et al., 2009). To obtain F_m_ and F_m_’, a saturating pulse light (630 nm, 8000 µmol photons m^−2^ s^−1^, 300 ms) was applied. Red light (630 nm, 400 µmol photons m^−2^ s^−1^) was supplied using a chip-on-board LED array. The values of Fv/Fm in the leaves of both the control- and HN-grown plants were shown in **Supplementary Table S1**. LET rate was calculated as follows: LET rate = α × Y(II) ×PFD. The value of α was supposed to be 0.42.

The signals for oxidized P700 (P700^+^), oxidized plastocyanin (PC^+^), and reduced ferredoxin (Fd^-^) were calculated based on the deconvolution of four pulse-modulated dual-wavelength difference signals in the near-infrared region (780 – 820, 820 – 870, 840 – 965, and 870–965 nm) (Klughammer and Schreiber, 2016). Both P700 and PC were completely reduced, and Fd was fully oxidized in the dark. To determine the total photo-oxidizable P700 (P700max) and PC (PCmax), a saturation flash was applied after 10 s of illumination with far-red light (740 nm). The following formulas were used: The quantum yield of oxidized P700 (P700^+^), Y(ND) = P700^+^/P700max. In the present research, we showed Y(ND) as P700^+^. Total photo-reducible Fd (Fdmax) was determined by illumination with red light (450 µmol photons m^−2^ s^−1^) after plant leaves were adapted to the dark for 5 min. The redox states of both P700 and PC under light illumination were evaluated as the ratios of P700^+^ and PC^+^ to total P700 and total PC, respectively. The redox state of Fd was also determined similarly. The values of P700max, PCmax, and Fdmax in the leaves of both the control- and HN-grown plants were shown in **Supplementary Table S1**.

For the analysis of dark-interval relaxation kinetics (DIRK analysis) (Sacksteder and Kramer, 2000), the red light was temporarily turned off for 400 ms at steady-state photosynthesis. The oxidation rate of Fd^-^ was estimated by a Dual/KLAS-NIR spectrophotometer and expressed as a relative value by estimating the initial decay of Fd^-^. The detected signal was confirmed as reduced Fd by comparison with a mutant expressing the flavodiiron protein (FLV) in *Arabidopsis thaliana* (Yamamoto et al., 2016), which showed the higher rate of the reduced Fd (**Supplementary Figure S1**). FLV reduces O_2_ to H_2_O using Fd as the electron donor (Sétif et al., 2020). vFd(CET) was estimated as follows; if only LET is driving the vFd, then the light dependence of the vFd should follow LET rate. In other words, vFd independent LET reflects the vFd(CET). In the low light intensities, as vFd(CET) is considered to have little function, the vFd was plotted to match the LET rate, and the vFd produced by the LET was then estimated from the graph. The gap between the vFd obtained from the analysis and estimated from the LET rate was then used as the vFd(CET). The other set different from the above set were used for the simultaneous analysis of the electrochromic shift (ECS) signal with CO_2_/H_2_O-exchange, as follows.

### Simultaneous measurements of electrochromic shift signal with CO_2_/H_2_O-exchange

Electrochromic shift (ECS) signal with CO_2_/H_2_O-exchange in sunflower plants (*Helianthus annuus*) were simultaneously analyzed by the method reported in the previous study (Maekawa et al., 2024). The magnitude of the ECS signal was analyzed by DIRK analysis (Sacksteder and Kramer, 2000; Avenson et al., 2004) and normalized as follows (Klughammer et al., 2013). A single turnover flash (50 μs) was used to illuminate the leaf for the single turnover (ST) flash-induced ECS signal (ECS_ST_). Then, the measured ECS signal was divided by ECS_ST_ and was used as proton motive force (pmf) (Shimakawa and Miyake, 2021). The contribution of both ΔpH and ΔΨ to the total ECS signal was separately evaluated after turning off the AL illumination over longer periods of darkness (Cruz et al., 2004).

### Statistical analytics

Statistical analysis of the Welch’s t-test was performed using the commercial software JMP8 (ver. 14.2.0, SAS Institute Inc., Cary, NC, USA).

## Results

**Figure 1:**
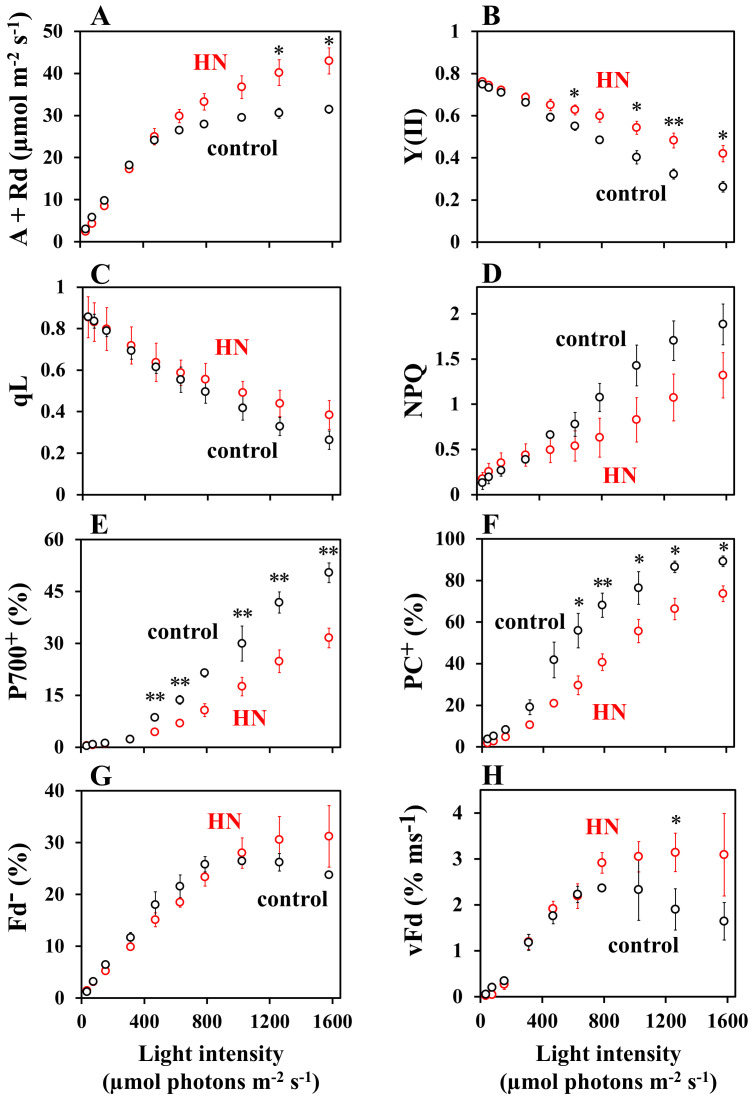
The impact of light intensity on photosynthesis parameters. **(A)** The gross CO_2_ assimilation rate, A + Rd; **(B)** The effective quantum yield of PSII, Y(II); **(C)** The redox state of plastoquinone, qL; **(D)** Non-photochemical quenching of Chl fluorescence, NPQ; **(E)** The oxidation state of P700, P700^+^; **(F)** the oxidation state of plastocyanin, PC^+^; **(G)** the reduced state of ferredoxin, Fd^-^; **(H)** the oxidation rate of Fd^-^, vFd in sunflower plants. The net CO_2_ assimilation rates **(A)** were measured concurrently with the other parameters under atmospheric conditions (40 Pa CO_2_, 21 kPa O_2_). The dark respiration rates (Rd) were measured prior to the commencement of light illumination. Once the net CO_2_ assimilation reached a steady state at a light intensity of 800 μmol photons m^-2^ s^-1^, the intensity was decreased to 50 and then increased to 1,600 in a sequential manner, following each attainment of a new steady-state CO_2_ assimilation. The gross CO_2_ assimilation rates are expressed as A + Rd. Each parameter is plotted against the light intensity. The black symbols represent data from the control-grown plants (n = 3), while the red symbols represent data from HN-grown plants. Data are means ± SD (n = 3). Where not visible, error bars are smaller than the symbols. *p< 0.05; **p< 0.01 (Welch’s t-test).

The dependence of the parameters: CO_2_ assimilation rate, chlorophyll (Chl) fluorescence [Y(II), qL, NPQ] and the redox states of P700, PC, and Fd on light intensity were examined in sunflower leaves grown in both the control- and high-nitrogen (HN) conditions (**Figure 1**). In control-grown conditions, the CO_2_ assimilation rate reached a maximum at light intensities of approximately 800 – 1,000 µmol photons m^-2^ s^-1^. In contrast, CO_2_ assimilationrates by HN-grown leaves increased with light intensity, even up to 1,600 µmol photons m^-2^ s^-1^ where they were higher than the control-grown leaves (**Figure 1A**). The value of Y(II) decreased with increasing light intensity in both the control- and the HN-grown leaves, but the Y(II) values were higher in the HN-grown leaves (**Figure 1B**). Similarly, the qL decreased with increasing light intensity in both conditions, with higher values observed in HN-grown leaves (**Figure 1C**). The qP also showed the similar response to the light intensity in the control- and HN-grown leaves (**Supplementary Figure S2**). Both the control- and the HN-grown leaves demonstrated an increase in NPQ with increasing light intensity, with the HN-grown leaves exhibiting lower NPQ values than the control (**Figure 1D**). For both growth conditions, P700^+^ demonstrated an increase with light intensity; however, HN-grown leaves exhibited lower P700^+^ levels than the control-grown leaves at higher light intensities (**Figure 1E**). Similarly, PC^+^ increased with light intensity in both growth conditions, with HN-grown leaves exhibiting lower PC^+^ levels than the control, indicating a more reduced redox state (**Figure 1F**). Both the control- and HN-grown leaves demonstrated an increase in Fd^-^ with increasing light intensity. In control-grown condition, the Fd^-^ reached a maximum at light intensities of approximately 800 – 1,000 µmol photons m^-2^ s^-1^, similar to the CO_2_ assimilation rate. In contrast, the Fd^-^ in HN-grown leaves maintained the maximum level, even up to 1,600 µmol photons m^-2^ s^-1^ (**Figure 1G**). Both the control- and HN-grown leaves demonstrated an increase in the oxidation rate of Fd^-^ (vFd) with increasing light intensity. The vFd was estimated using DIRK analysis, as described in MATERIALS AND METHODS section. In control-grown conditions, vFd reached a maximum at the light intensity of 800 – 1,000 µmol photons m^-2^ s^-1^, similar to Fd^-^ (**Figure 1H**). In contrast, the HN-grown leaves maintained the maximum rate, even up to 1,600 µmol photons m^-2^ s^-1^.

**Figure 2:**
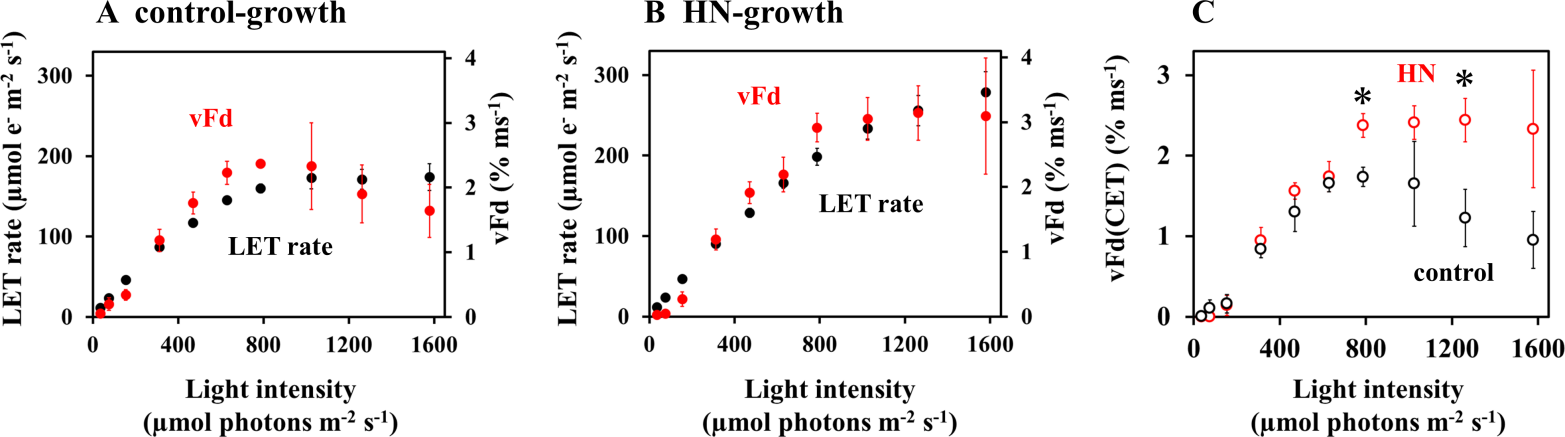
The effects of light intensity on photosynthesis parameters, including the linear electron transport (LET) rate, and the oxidation rate of Fd^-^ (vFd), were investigated in sunflower plants. The both LET rate and vFd were estimated from data presented in **Figure 1**, as detailed in the materials and methods section. Both LET rate and vFd are plotted against the light intensity. **(A)** control-grown plants; **(B)** HN-grown plants. The black symbols represent the LET rate, while the red symbols represent vFd. **(C)** vFd(CET) were plotted against the light intensity. The black symbols represent data from the control-grown plants, while the red symbols represent data from HN-grown plants. Data are means ± SD (n = 3). Where not visible, error bars are smaller than the symbols. *p< 0.05 (Welch’s t-test).

The trend of vFd values over light intensity in control-grown leaves clearly showed extra electron transport catalyzed by Fd, other than LET to CO_2_ assimilation. Both vFd and LET rate were then plotted against light intensity (**Figure 2**). At low light intensities (50 µmol photons m^-2^ s^-1^), vFd was minimal (**Figure 1**), indicating that the PSI-CET rate can be considered negligible (Ohnishi et al., 2023; Maekawa et al., 2024). The values were plotted in order to compare vFd in low-light conditions with the LET rate. In both the control- and HN-grown leaves, LET rate increased with light intensity (**Figures 2A, B**), but unlike vFd, rates of LET did not show a decrease at higher light intensities. A light-saturated rate of LET in the control-grown leaves was observed at 800 – 1,000 µmol photons m^-2^ s^-1^, while HN-grown leaves did not saturate even at 1,600 µmol photons m^-2^ s^-1^, a phenomenon that mirrored the CO_2_ assimilation rates (**Figure 1**).

The PSI-CET rate was estimated according to the following methodology: If the LET rate and vFd exhibited an identical light intensity dependence, then PSI-CET was deemed to be inactive (Ohnishi et al., 2023; Maekawa et al., 2024). Conversely, if vFd exhibited a more pronounced response, this increase indicated active PSI-CET, vFd(CET), which is represented by a shaded area (**Supplementary Figure S3**). In the control-grown leaves, which show a decrease in vFd at the highest light intensities, the extra electron transport catalyzed by Fd relative to LET indicates the existence of PSI-CET. As for the control-grown leaves, HN-grown leaves showed vFd additional to that required for LET, demonstrating the existence of PSI-CET. In both the control- and HN-grown leaves, vFd(CET) was plotted against the light intensity (**Figure 2C**). In the control-grown leaves, the activity of vFd(CET) over increasing light intensity was found to mirror the increase and decrease in overall vFd (**Figure 1H**), indicating that variable vFd(CET) is responsible for the difference between control- and HN-grown plants.

**Figure 3:**
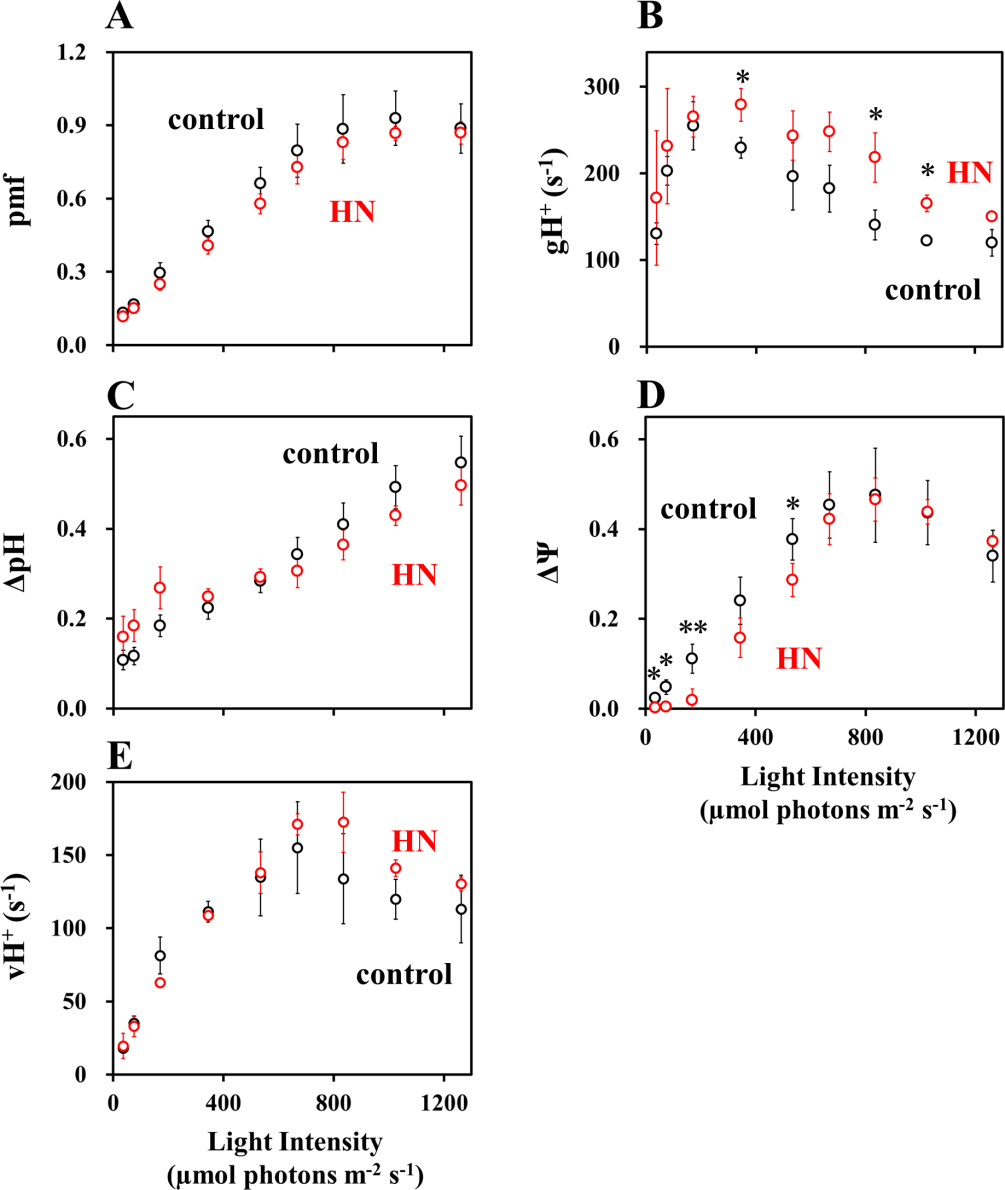
The impact of light intensity on photosynthesis parameters: **(A)** proton motive force (pmf); **(B)** gH^+^; **(C)** ΔpH; **(D)** ΔΨ; **(E)** vH^+^ in sunflower plants. These parameters were measured under atmospheric conditions (40 Pa CO_2_, 21 kPa O_2_), as detailed in the materials and methods section. Each parameter is plotted against the light intensity. The black symbols represent control-grown plants, and the red symbols represent HN-grown plants. Data are means ± SD (n = 3). Where not visible, error bars are smaller than the symbols. *p< 0.05; **p< 0.01 (Welch’s t-test).

To elucidate the physiological role of PSI-CET, a correlation analysis was conducted between vFd(CET) and proton motive force (pmf). The dependence of the proton motive force (pmf) and its components, ΔpH and ΔΨ, on light intensity was evaluated using ECS analysis (**Figure 3**). In both the control- and HN-grown leaves, the dependence of pmf on light intensity was analyzed (**Figure 3**). It is noteworthy that the pmf in HN-grown leaves was observed to be slightly (but not significantly) lower than that in the control-grown leaves. This could be due to the higher consumption rate of ATP required to support the elevated CO_2_ assimilation observed in HN-grown leaves relative to that in the control-grown leaves (**Figure 1A**). Such a model is supported by the significantly higher H^+^-conductance (gH^+^) seen in HN plants (**Figure 3B**). The gH^+^ depends on the catalytic activity of ATP synthase, and the recovery rates of its substrates, ADP and P_i_. A higher CO_2_ assimilation rate would regenerate ADP and P_i_ more rapidly, which contributes to the lower pmf (Miyake, 2020; Wada et al., 2020). In the control- and HN-grown leaves, ΔpH continued to increase with light intensity (**Figure 3C**). In contrast, both the control- and HN-grown leaves showed an increase of ΔΨ up to approximately 800 µmol photons m^-2^ s^-1^, followed by a light dependent decrease in ΔΨ (**Figure 3D**), mirroring the response in vFd(CET). Interestingly, the increase in ΔΨ was significantly slower in HN plants, and the decrease of ΔΨ at light intensities above 800 µmol photon m^-2^ s^-1^ was less pronounced relative to control-grown leaves. These responses of ΔΨ to increasing light intensity correspond to those in vFd(CET) (**Figure 2C**) and might suggest that PSI-CET, as represented by vFd(CET), plays a role in CO_2_ assimilation related to the development of greater ΔΨ. Furthermore, the dependence of the rate of pmf utilization (vH^+^ = gH^+^ × pmf) on light intensity were compared between the control- and HN-grown leaves (**Figure 3E**). In the both leaves, showed an increase of vH^+^ up to approximately 800 µmol photons m^-2^ s^-1^, followed by a light dependent decrease in vH^+^. The extent of the decrease in vH^+^ in the control-grown leaves was larger than that in the HN-grown leaves (**Figure 3E**). These responses of vH^+^ to increasing light intensity also correspond to those in vFd(CET) (**Figure 2C**). LET rate increased with light intensity (**Figures 2A, B**), but unlike vH^+^, LET rate did not show a decrease at higher light intensities. Only LET could not support the ATP usage in CO_2_ assimilation. That is, PSI-CET would support the ATP usage by the development of pmf with LET.

**Figure 4:**
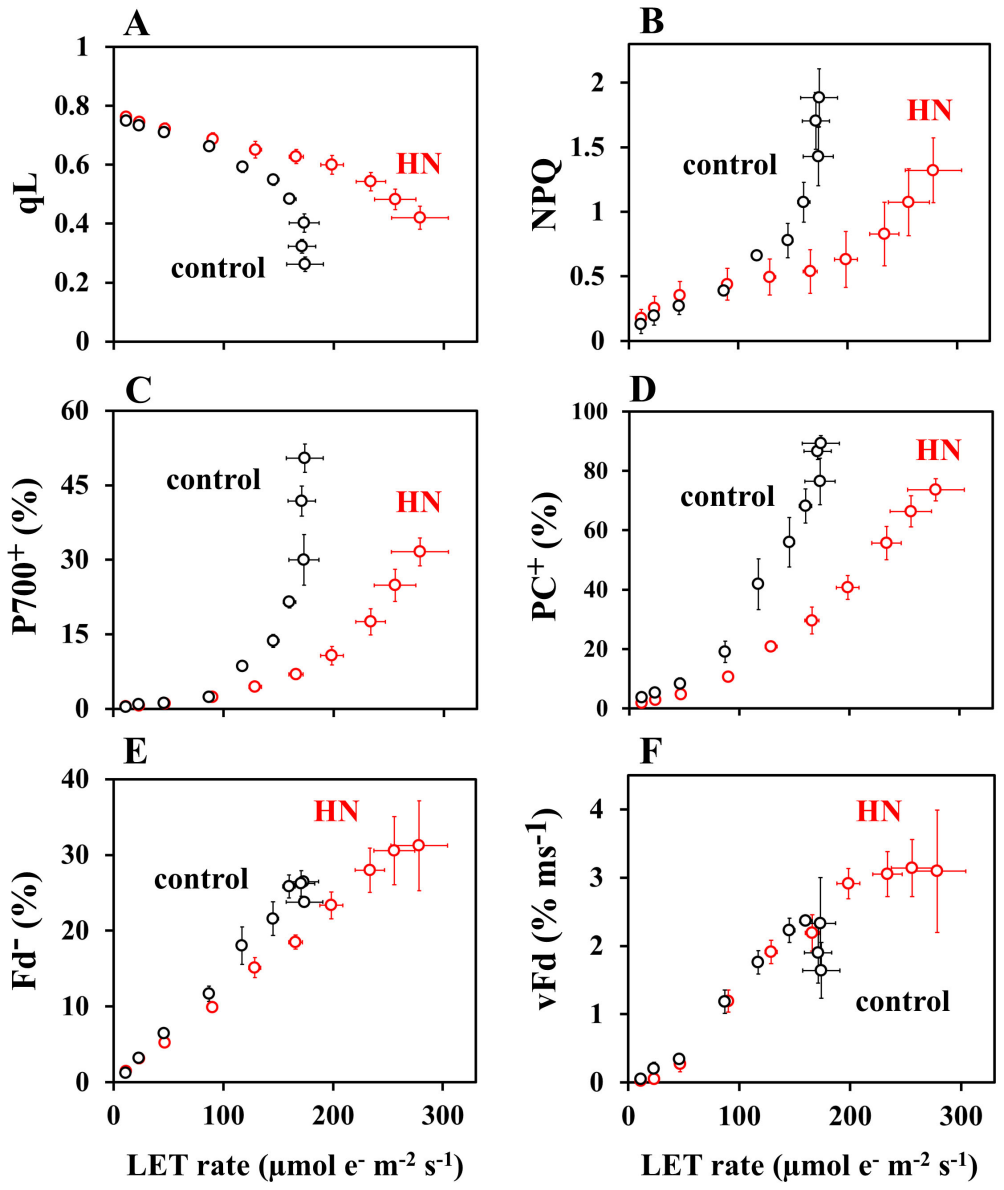
The dependence of photosynthesis parameters: **(A)** qL; **(B)** NPQ; **(C)** PC^+^; **(D)** P700^+^; **(E)** Fd^-^; **(F)** vFd on LET rate in sunflower plants. These parameters were from **Figures 1** and **2**. Each parameter is plotted against LET rate. The black symbols represent data from the control-grown plants, while the red symbols represent data from HN-grown plants. Data are means ± SD (n = 3). Where not visible, error bars are smaller than the symbols.

**Figure 5:**
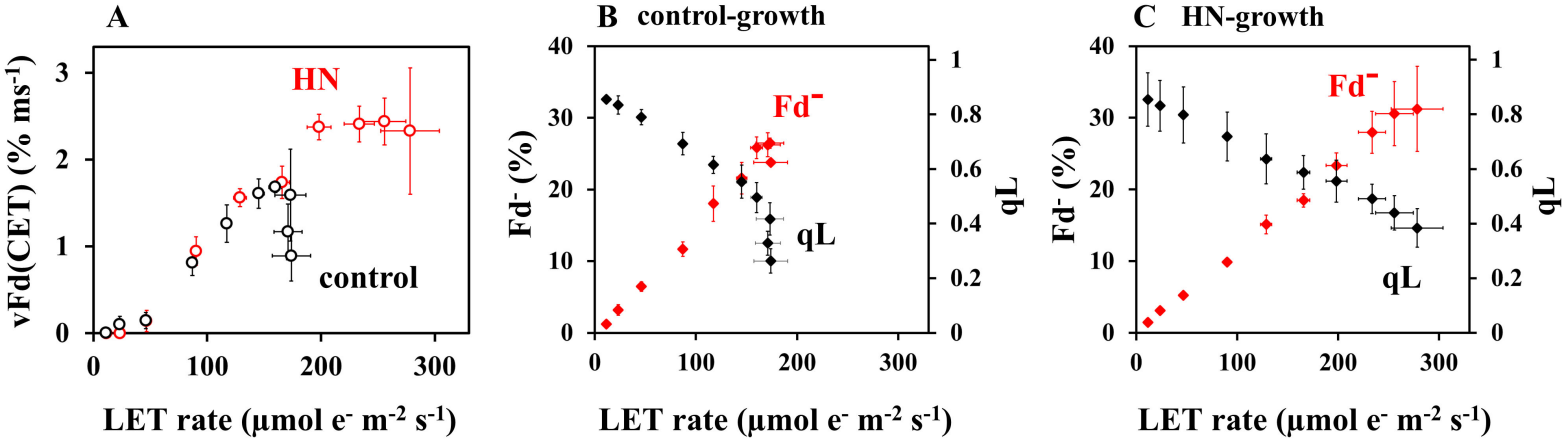
The impacts of the redox state of Fd and PQ on vFd(CET). **(A)** vFd(CET) was from **Figure 2**, and plotted against LET rate. The black symbols represent data from the control-grown plants, while the red symbols represent data from HN-grown plants. **(B)** Both Fd^-^ and qL were from **Figures 1** and **2**, and plotted against LET rate in the control-grown plants. The black symbols represent data from the control-grown plants, while the red symbols represent data from HN-grown plants. **(C)** Both Fd^-^ and qL were from **Figures 1** and **2**, and plotted against LET rate in the HN-grown plants. The black symbols represent data from the control-grown plants, while the red symbols represent data from HN-grown plants. Data are means ± SD (n = 3). Where not visible, error bars are smaller than the symbols.

To elucidate what causes the rise and then fall in vFd(CET) over increasing light intensity (**Figure 2C**), we analyzed the responses of PSII and PSI acceptors and donors to rates of LET (**Figure 4**). In all plants, increasing LET rates correlated with decreasing qL (proportional to acceptor availability at PSII), increasing protective non-photochemical quenching (NPQ), increasing oxidation of PSI (P700^+^) and PC (PC^+^) (**Figures 4B–D**). In all cases, saturation of LET above around 100 µmol e^-^ m^-2^ s^-1^ in control-grown plants correlates with divergence from HN-grown plants values of qL, NPQ, P700^+^ and PC^+^, where changes are attenuated. Saturation of LET correlated exactly with saturation of Fd^-^ in control-grown plants, while neither parameter reached saturation in HN-grown plants (**Figure 4E**). In both control- and HN-grown leaves, vFd increased with LET (**Figure 4F**), but control-grown leaves showed a downregulation of vFd as the LET rate saturates. By contrast, HN-grown leaves maintained vFd, as LET rates increased further. The relationship between LET and vFd is reflected in that between LET and vFd(CET) (**Figure 5A**), with a drop in vFd(CET) as LET rates saturate. This saturation also correlates with a drop in qL values (**Figure 4A**), presumably because PSI-CET requires both the electron donor Fd^-^, and the electron acceptor PQ to catalyze the electron transport around PSI. Fd^-^ and PQ availability (qL) were then plotted against LET rate (**Figure 5**). In the control-grown leaves, as LET saturates, Fd^-^ also saturates at about 25%, while qL drops from 0.5 to 0.25 (50%) (**Figure 5B**). In HN-grown leaves, Fd^-^ continued to increase to about 30%, and qL continued to decrease (**Figure 5C**). These results strongly indicate that vFd(CET) in the leaves adheres to the PSI-CET functional model proposed by Allen (2003). In the control-grown leaves, as LET increases, Fd^-^ levels rise, stimulating PSI-CET until it is suppressed by limited PQ availability (**Figures 5A, B**). By contrast, in HN-grown leaves, vFd(CET) does not drop at high LET (**Figure 5A**) due to a more gradual decline in qL levels relative to LET (**Figures 5A, C**).

As described above, PSI-CET rate was evaluated as vFd(CET), and the data indicates it is regulated by the redox state of both Fd and PQ. These results strongly suggest the existence of a mediator to catalyze PSI-CET. The rate of PSI-CET as proposed by (Allen, 2003), is expressed as:


(1) $$\text{vFd}\left(\text{CET}\right)=\text{k}\times {\text{Fd}}^{-}\times \text{PQ}$$

**Figure 6:**
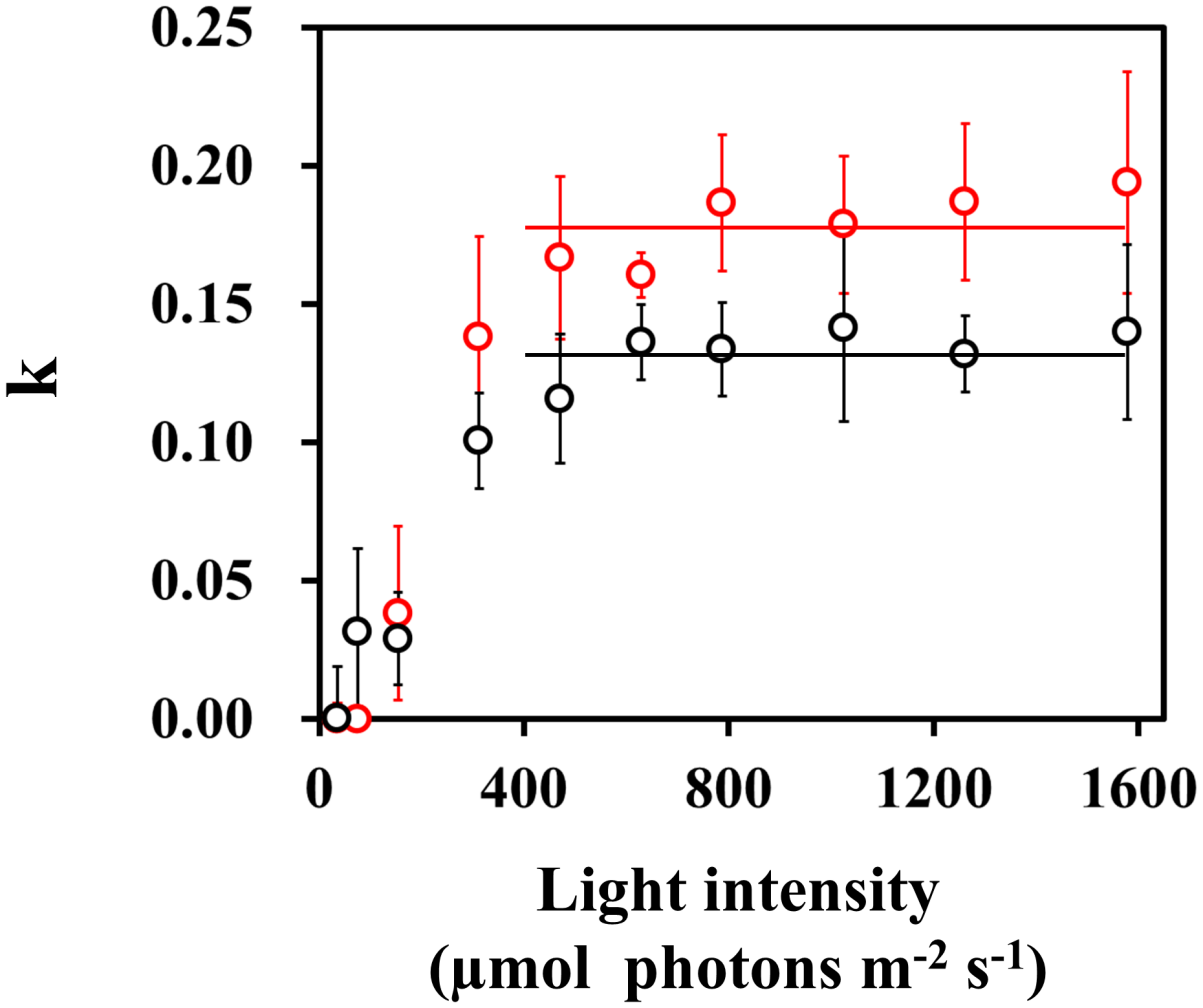
The dependence of the catalytic constant k in PSI-CET on the light intensity. The k value was estimated from the values of qL, Fd^-^ and vFd(CET) from **Figure 5**, following the Equation 1: k = vFd(CET)/(qL x Fd^-^). The estimated k values were plotted against the light intensity. Both black and red horizontal lines: the mean values of k, 0.170 and 0.129 in control- and HN-grown leaves.

The constant k depends on the catalytic rate and the abundance of a PSI-CET mediator, and can be estimated from the data in **Figures 1** and **2**. From Equation 1, the k value was calculated using the parameters; qL as oxidized PQ availability and Fd^-^ as reduced Fd availability (**Figure 6**). These k values increased with light intensity and above 350 µmol photons m^-2^ s^-1^, k values reached values for HN-grown leaves (0.170 ± 0.019), significantly higher than for control-grown leaves (0.129 ± 0.015) (*p*< 0.01) (**Figure 6**).

## Discussion

The present research explores the mechanism and physiological function of PSI-CET *in vivo*. It was observed that over increasing light intensity both the PQ and Fd redox states altered in intact sunflower leaves. These experimental conditions were therefore judged conducive to experiments into how rates of PSI-CET are regulated *in vivo*. A more reduced state of Fd increased rates of PSI-CET while a more reduced state of PQ decreased rates of PSI-CET (**Figure 5**). In other words, the maximum rate of PSI-CET was found to be dependent on the redox state of both PQ (the end acceptor) and Fd (the electron donor). At higher light intensities, control of PSI-CET was dominated by the reduction state of PQ, with Fd remaining at a constantly reduced level around 25% (**Figure 5**). In comparison to the control-grown plants, the HN-grown plants exhibited a higher rate of PSI-CET at higher light, presumably due to the more oxidized state of PQ relative to LET in these plants (**Figure 4**).

This study provides key insight into the physiological function of PSI-CET. It has long been proposed that PSI-CET, in contrast to LET, contributes to the generation of ATP by supplying proton motive force (pmf) without producing NADPH (Heber and Walker, 1992; Yamori and Shikanai, 2016). Therefore, if the PSI-CET rate (vCET), which serves as an indicator of its activity, correlated with parameters associated with pmf—namely ΔpH, ΔΨ, gH^+^, and vH^+^—this would provide circumstantial evidence supporting the physiological significance of PSI-CET. We observed a positive correlation between vCET and ΔΨ (**Figures 1**–**3**), and similarly between vCET and vH^+^ (**Figures 4**, **5**). At this point, however, it must be noted that the presence of a ΔΨ component has been challenged in the literature (Wilson et al., 2021). In *Arabidopsis* mutants lacking non-photochemical quenching (NPQ) capacity, ΔΨ formation is reportedly suppressed (Wilson et al., 2021). Likewise, in the mutants deficient in zeaxanthin or lutein—key pigments in the xanthophyll cycle—ΔΨ formation is impaired. Ruban group argued that pmf reflects ΔpH only (Wilson et al., 2021). Our own findings raise similar concerns regarding the validity of ΔpH and ΔΨ estimation using the methods developed by the Kramer group (**Figure 3**). We observed that ΔpH did not saturate with increasing light intensity, which cannot be explained by the saturating LET rate or the peaked response of vCET. These findings underscore the need for careful interpretation of ΔpH and ΔΨ measurements. Therefore, we focused on alternative pmf-related parameters—specifically pmf, gH^+^, and especially vH^+^ (**Figures 3**–**5**). Notably, vH^+^ exhibited a peaked response to light intensity, similar to vFd, which encompasses vCET. Moreover, vH^+^, vFd, and vCET all showed comparable dependencies on the LET rate. These findings strongly support the conclusion that PSI-CET contributes to the formation of pmf and thereby promotes ATP synthesis.

As shown in **Figure 6**, we confirmed the existence of the PSI-CET mediator, ferredoxin quinone oxidoreductase (FQR). So far, ferredoxin NADP oxidoreductase (FNR), NADH dehydrogenase (NDH), cytochrome (Cyt) *b*_6_/*f* complex, and pgr5/pgrL1 have been proposed as PSI-CET mediators. However, it has already been clarified that NDH and pgr5/pgrl1 are not involved in the major pathway of PSI-CET in higher plants (Ohnishi et al., 2023; Maekawa et al., 2024). In cyanobacteria, NDH is the major mediator of PSI-CET, and the PSI-CET rate catalyzed by NDH is comparable to the LET rate (Hu et al., 2013; Han et al., 2017; Theune et al., 2021; Hualing, 2022; Liu et al., 2024). In higher plants, however, the amount of NDH is very low compared to other components of the thylakoid membrane electron transport chain. For example, in *Arabidopsis thaliana*, the amount of NDH is approximately 1% of that of PSI (Pribil et al., 2014; Johnson, 2025). This likely explains why the NDH-dependent PSI-CET rate is low in higher plants. The involvements of FNR and Cyt *b*_6_/*f* complex as FQR in PSI-CET must be clarified in the future. However, FNR does not interact with Cyt *b*_6_/*f* complex (Zakharov et al., 2022). Furthermore, we found an increase of the k value with light intensity, until it reached a constant level above 400 µmol photons m^-2^ s^-1^. These facts demonstrate the activation of this FQR pathway.

How is the PSI-CET functional expression mode revealed in this study related to the light-dependent structural reorganization of thylakoid membranes that underpins the functional expression of PSI-CET and LET (Garty et al., 2024)? When *Arabidopsis* leaves that have been kept in darkness are transferred to light, part of the grana with appressed thylakoid membranes is transformed into stacked thylakoid doublets possessing properties characteristic of the non-appressed stroma thylakoid membranes. This structural change is reversible: phosphorylation of PSII–LHCII complexes in grana thylakoids under illumination induces their transition into stacked thylakoid doublets, while dephosphorylation triggers the return to grana structures. The appressed thylakoid membranes are enriched in PSII and LHCII, whereas the non-appressed stroma thylakoid membranes are enriched in PSI, ATP synthase, and the Cyt b_6_/f complex (Jennings et al., 1979; Andersson and Anderson, 1980; Albertsson, 2001; Danielsson et al., 2004; Caffarri et al., 2014; Kirchhoff et al., 2017). This physical separation of PSI and PSII is thought to suppress plastoquinone (PQ) diffusion and thereby promote PSI-CET (Garty et al., 2024). As illumination continues, the appearance of stacked thylakoid doublets reduces the physical distance between PSI and PSII, which has been proposed to promote LET activity (Garty et al., 2024). As shown in **Figures 4** and **5**, in control-grown plants, PSI-CET rates decreased when light intensity increased beyond the saturation point of the LET rate. However, compared to control-grown plants, HN-grown plants maintained a more oxidized PQ pool and a more reduced Fd pool even at higher light intensities. Consequently, PSI-CET rates remained high in HN-grown leaves under strong light. At first glance, this appears contradictory to the notion that the formation of stacked thylakoid doublets under illumination suppresses PSI-CET. It is possible that PSI-CET activity is strongly influenced by the redox states of PQ and Fd. The relationship between the formation of stacked thylakoid doublets and the PSI-CET functional expression model (Equation 1) remains an intriguing question and warrants future investigation.

Although a more reduced state of Fd was necessary for the activation of PSI-CET, Fd^-^ (%) reached a plateau in response to increasing the light intensity (**Figures 1**, **2**). In the control-grown plants, the Fd^-^ (%) reached a plateau at approximately 800 − 1,000 µmol photons m^-2^ s^-1^ (**Figure 1**). A constant redox state of Fd is contingent upon equilibrium between the rates of reduction and oxidation of Fd. The oxidation rate of Fd was saturated, as evidenced by the saturation of both the CO_2_ assimilation rate and the LET rate (**Figures 1**, **2**). This indicates that the photorespiration rate also reached a saturation point by approximately 800 – 1,000 µmol photons m^-2^ s^-1^ (Sejima et al., 2016; Wada et al., 2020). Despite this saturation of electron sink activity, the redox state of Fd remained largely unaltered with increasing light intensity. That is, the reduction rate of Fd should be downregulated. The acidification of the luminal side of thylakoid membranes has been demonstrated to reduce the activity of PQH_2_ oxidation by the Cyt *b*_6_/*f* complex (Tikhonov, 2014; Degen and Johnson, 2024). However, the pmf reached a saturation point in response to further increases in light intensity above approximately 900 µmol photons m^-2^ s^-1^. In other words, if the pmf reflected ΔpH mainly (Wilson et al., 2021), the acidification control of PQH_2_ oxidation by the Cyt *b*_6_/*f* complex was not operational at higher light intensities, although the increase in light intensity resulted in the further oxidation of P700 in PSI. It can be inferred that an alternative regulatory mechanism of PQH_2_ oxidation is responsible for suppressing the electron flow from PQH_2_ to PSI. We have put forth a hypothesis regarding the suppression mechanism of PQH_2_ oxidation, reduction-induced suppression of electron flow, RISE (Shaku et al., 2016; Shimakawa et al., 2018; Furutani et al., 2020; Malone et al., 2021; Wada et al., 2020; Degen and Johnson, 2024; Johnson, 2025). A higher reduced state of PQ inhibits the Q cycle in the Cyt *b*_6_/*f* complex, resulting in the oxidation of P700 in PSI. Indeed, the reduction of PQ persisted even when the light intensity was augmented above approximately 800 µmol photons m^-2^ s^-1^ (**Figure 1C**). It is conceivable that RISE could serve to maintain a constant reduction level of Fd at higher light intensities in the control-grown leaves. Conversely, both luminal acidification and RISE would act in concert to inhibit the further reduction of Fd at the higher light intensity in HN-grown leaves. The pmf increase and reduction of PQ persisted at the light intensities exceeding 800 µmol photons m^-2^ s^-1^ (**Figures 1**, **5**).

The suppression of electron transport from PQH_2_ to the PSI acceptor side by both luminal acidification and RISE indicates that the supply of both NADPH and ATP necessary for the dark reaction is sufficient. Furthermore, this demonstrates that PSI should be protected from photoinhibition under the higher light intensity. The accumulation of electrons at the acceptor side of PSI, and the reduction of electron carriers: A_0_, A_1_, F_X_, and F_A_/F_B_ in the PSI complex elevates the likelihood of the carriers’ reaction with O_2_, resulting in the production of O_2_^-^ (Asada, 1999; Khorobrykh et al., 2020; Miyake, 2020). Indeed, the experiment of filling the aforementioned electron carriers with electrons has been demonstrated to result in oxidative damage to PSI (Sejima et al., 2014; Furutani et al., 2023; Shimakawa et al., 2024). The oxidation of PSI, which is induced by both the acidification of the luminal side and RISE, has been demonstrated to suppress the photoinhibition of PSI by oxidizing the electron carriers on the acceptor side of PSI (Sejima et al., 2014; Shimakawa et al., 2016; Furutani et al., 2023). The aforementioned facts, in conjunction with the constant reduction in Fd levels, illustrate that the prevention of oxidative damage is of greater significance than PSI-CET.

Additionally, non-photochemical quenching (NPQ) of Chl fluorescence demonstrated a positive correlation with increasing light intensity in both the control- and HN-grown leaves (**Figure 1**). In general, the induction of NPQ requires the luminal acidification of thylakoid membranes, as observed in pmf formation (Baker et al., 2007; Ruban et al., 2012; Suzorsa et al., 2016). As previously outlined, the pmf reached a saturation point at approximately 800 µmol photons m^-2^ s^-1^, particularly in the control-grown leaves (**Figure 3A**). Nevertheless, the NPQ continued to increase with increasing light intensity. The various Chl fluorescence parameters are interrelated and none can be considered independent (Miyake et al., 2009). Consequently, alterations in the remaining parameters will result in a corresponding change in NPQ, even when the pmf remains constant (Miyake et al., 2009; Ruban and Murche, 2012). The relationship between these parameters in Chl fluorescence, as observed in both the lake and puddle models, can be described as follows: In the lake model, NPQ is defined as follows: NPQ = [1/Y(II)] × {qL × [1-Y(II)] × [(Fv/Fm)/(1-Fv/Fm)]} - 1 (Miyake et al., 2009); in the puddle model, NPQ is given by the following equation: NPQ = [1/Y(II)] × qP × [(Fv/Fm)/(1-Fv/Fm)] - [1 + (Fv/Fm)/(1-Fv/Fm)] (Ruban and Murche, 2012). In the puddle model, qP represents the parameter for photochemical quenching of Chl fluorescence (Oxborough and Baker, 1997; Schreiber et al., 1986). The continuation of the increase in NPQ will be the result of the continuation of the decrease in both Y(II) and qL (**Figure 1**).

**Figure 7:**
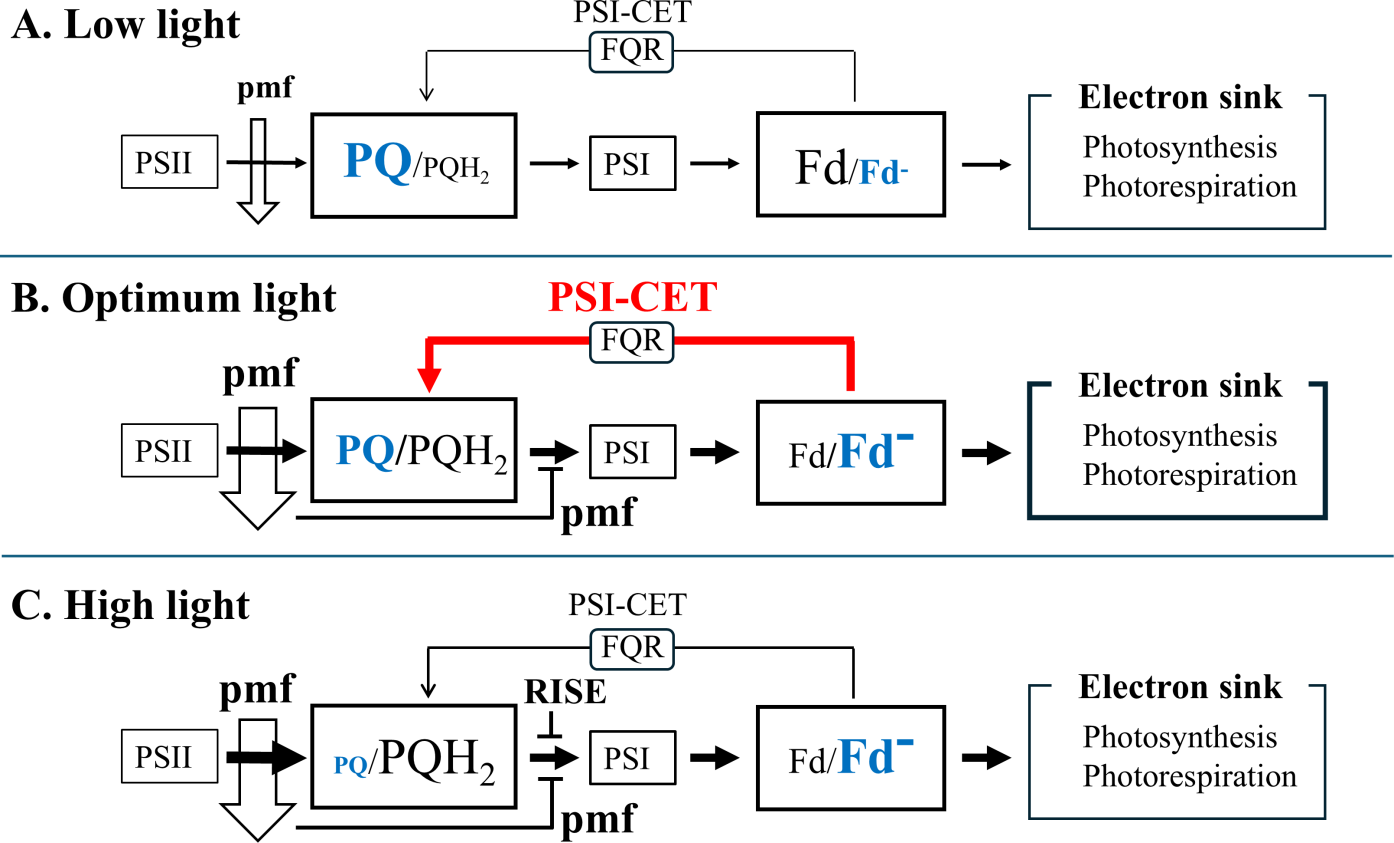
The expression model of PSI-CET in response to the light intensity: **(A)** Low light; **(B)** Optimum light; **(C)** High light. Please see the details in the discussion section.

The characteristics of PSI-CET, as elucidated in the present research, are summarized in **Figure 7**. Ferredoxin-quinone oxidoreductase (FQR) catalyzes PSI-CET. The expression of PSI-CET is contingent upon the redox state of both PQ and Fd, which in turn is responsive to changes in light intensity. In conditions of low light intensity, the rate of CO_2_ assimilation is limited by the availability of light energy. The ratios of both PQ/PQH_2_ and Fd/Fd^-^ are high. Subsequently, the rate of PSI-CET is found to be negligible in comparison to that of LET (**Figure 7A**). As light intensity is increased to a level at which CO_2_ assimilation is saturated, for example, approximately 800 – 900 µmol photons m^-2^ s^-1^, the rate of LET is observed to increase. Furthermore, the reduction of both PQ and Fd results in an increase in the PSI-CET rate to its maximum value (**Figure 7B**, Optimum light). The increase in both LET and PSI-CET rates results in the formation of the proton motive force (pmf). The acidification of the luminal side, as observed in the pmf, induces the oxidation of both PC and P700 (**Figure 1**). Furthermore, an additional increase in light intensity beyond the saturation point of the CO_2_ assimilation rate facilitates a greater reduction in PQ to a higher value of PQH_2_/PQ. Conversely, the reduction level of Fd remains unaltered (**Figure 7C**, High Light). The reduction of PQ has the effect of suppressing the PSI-CET rate. At high light, the highly reduced state of PQ induces RISE, which maintains the redox level of the electron carriers of the acceptor-side of PSI in the oxidized state, as indicated by the constant reduced state of Fd. This inhibits the production of reactive oxygen species (ROS) and safeguards PSI from oxidative damage (Furutani et al., 2023).

## Data Availability

The original contributions presented in the study are included in the article/**Supplementary Material**. Further inquiries can be directed to the corresponding author/s.

## Supplementary material

Supplementary Figure 1 Responses of Fd^-^ to actinic light illumination in WT and FLV overexpressed (*35S; PpFlv no.13*) in *Arabidopsis thaliana*. Actinic light illumination (200 µmol mol photons m^-2^ s^-1^, 3 s) to the leaves of WT **(A)** and *35S; PpFlv no.13* **(B)** reduced Fd. Dark periods are indicated with a black bar, light period is indicated with a white bar. Signals of the reduced Fd were monitored by DUAL/KLAS-NIR, as described in MATERIALS AND METHODS section.

Supplementary Figure 2 Dependence of qP on both **(A)** the light intensity and **(B)** LET rate. The values of qP were estimated from the data in **Figure 1** (Ruban and Murche, 2012). The black symbols represent the control-grown plants, while the red symbols represent the HN-grown plants. Where not visible, error bars are smaller than the symbols. *, *p*< 0.05 (Welch’s t-test).

Supplementary Figure 3 The effects of light intensity on photosynthesis parameters, including the linear electron transport (LET) rate, and the oxidation rate of Fd^-^ (vFd), were investigated in sunflower plants. The both LET rate and vFd were estimated from data presented in **Figure 1**, as detailed in the MATERIALS AND METHODS section. Both LET rate and vFd are plotted against the light intensity to align as described in the MATERIALS AND METHODS section. **(A)** control-grown plants; **(B)** HN-grown plants. The black symbols represent the LET rate, while the red symbols represent vFd. The shaded area depicts the rate of Fd-dependent cyclic electron transport around PSI, represented by vFd(CET) as shown in **Figure 2C**. Data are means ± SD (n = 3). Where not visible, error bars are smaller than the symbols.

Supplementary Table 1 Contents of chlorophyll (Chl), total nitrogen (N), and Rubisco, Fv/Fm, P700max, PCmax, and Fdmax in the leaves of sunflower plants. Contents of Chl, total nitrogen (N), and Rubisco were measured, as described in the MATERIALS AND METHODS section. Data are means ± SD (n = 3). **p*< 0.05, ***p*< 0.01 (Welch’s t-test).
