# Supplementary figures and images for: The regulation of PSI cyclic electron transport by both plastoquinone and ferredoxin redox states: correlation with the rate of proton motive force utilization
Source: Front Plant Sci. 2025 Aug 22;16:1626163. doi: 10.3389/fpls.2025.1626163 (PMC12427027; doi:10.3389/fpls.2025.1626163)

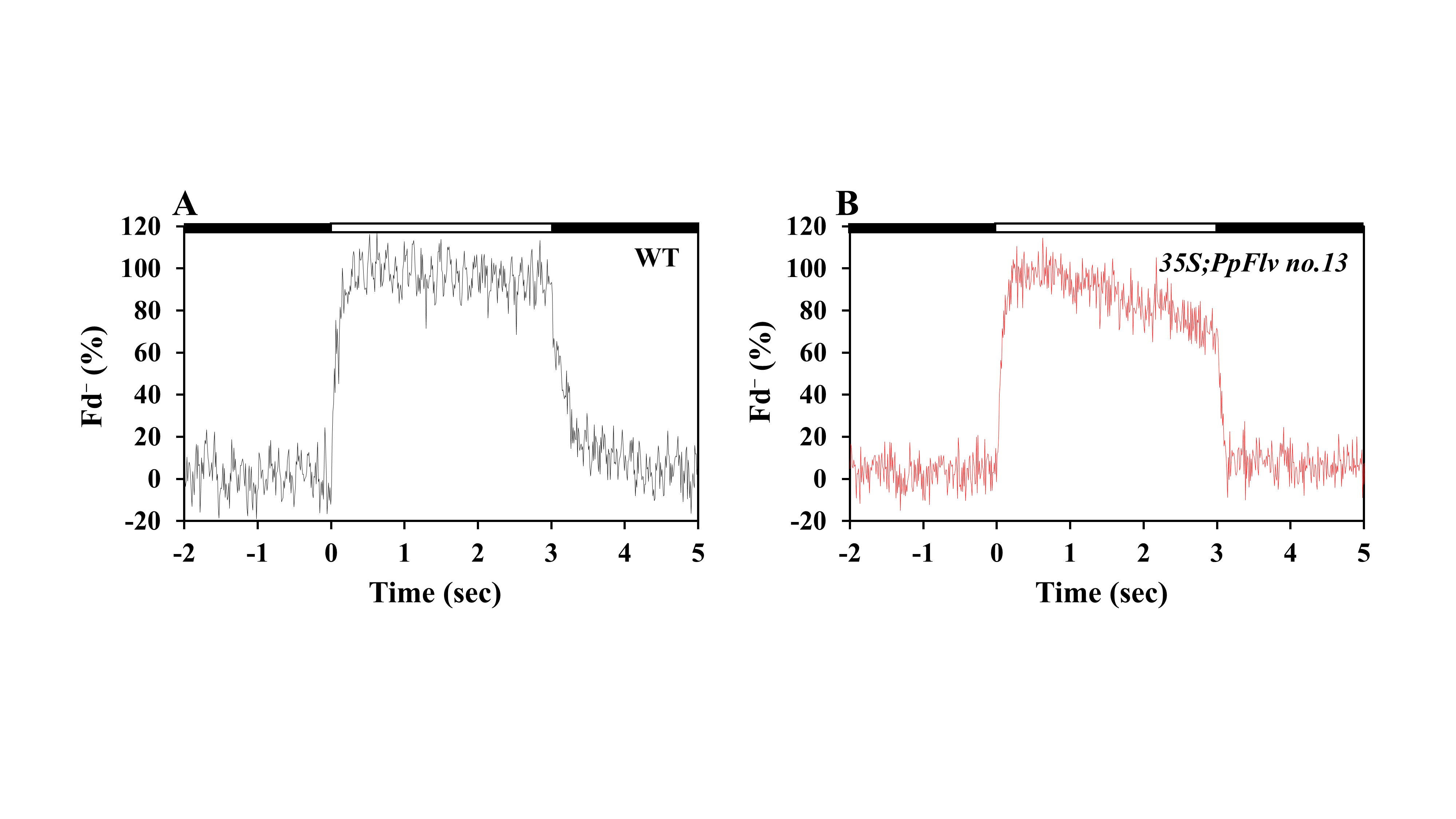

Supplement: Supplementary Figure 1 — Responses of Fd- to actinic light illumination in WT and FLV overexpressed (35S; PpFlv no.13) in Arabidopsis thaliana. Actinic light illumination (200 µmol mol photons m-2 s-1, 3 s) to the leaves of WT (A) and 35S; PpFlv no.13 (B) reduced Fd. Dark periods are indicated with a black bar, light period is indicated with a white bar. Signals of the reduced Fd were monitored by DUAL/KLAS-NIR, as described in MATERIALS AND METHODS section. [file DataSheet1.zip › Supplementary Figure S1.tif]

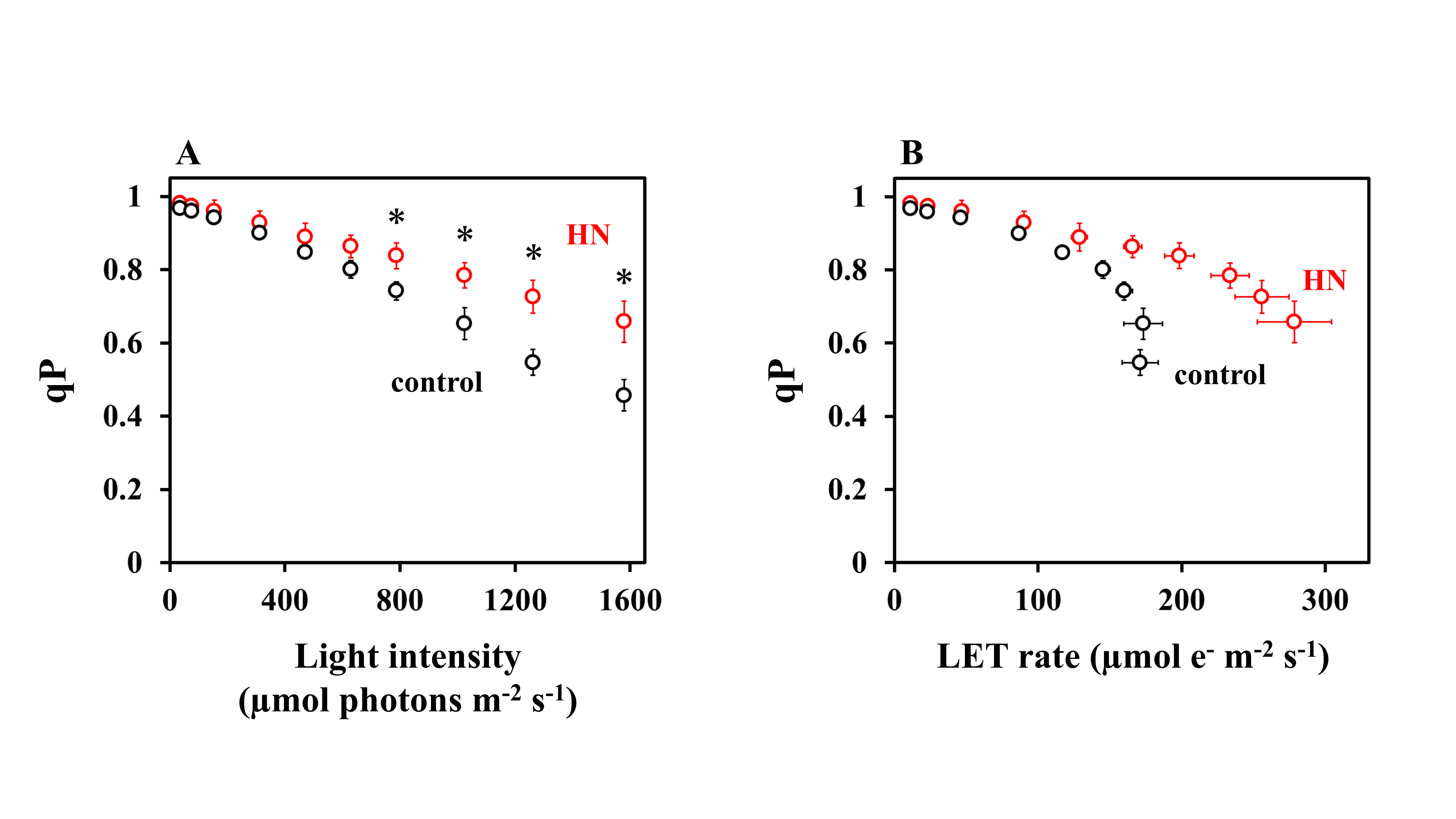

Supplement: Supplementary Figure 1 — Responses of Fd- to actinic light illumination in WT and FLV overexpressed (35S; PpFlv no.13) in Arabidopsis thaliana. Actinic light illumination (200 µmol mol photons m-2 s-1, 3 s) to the leaves of WT (A) and 35S; PpFlv no.13 (B) reduced Fd. Dark periods are indicated with a black bar, light period is indicated with a white bar. Signals of the reduced Fd were monitored by DUAL/KLAS-NIR, as described in MATERIALS AND METHODS section. [file DataSheet1.zip › Supplementary Figure S2.tif]

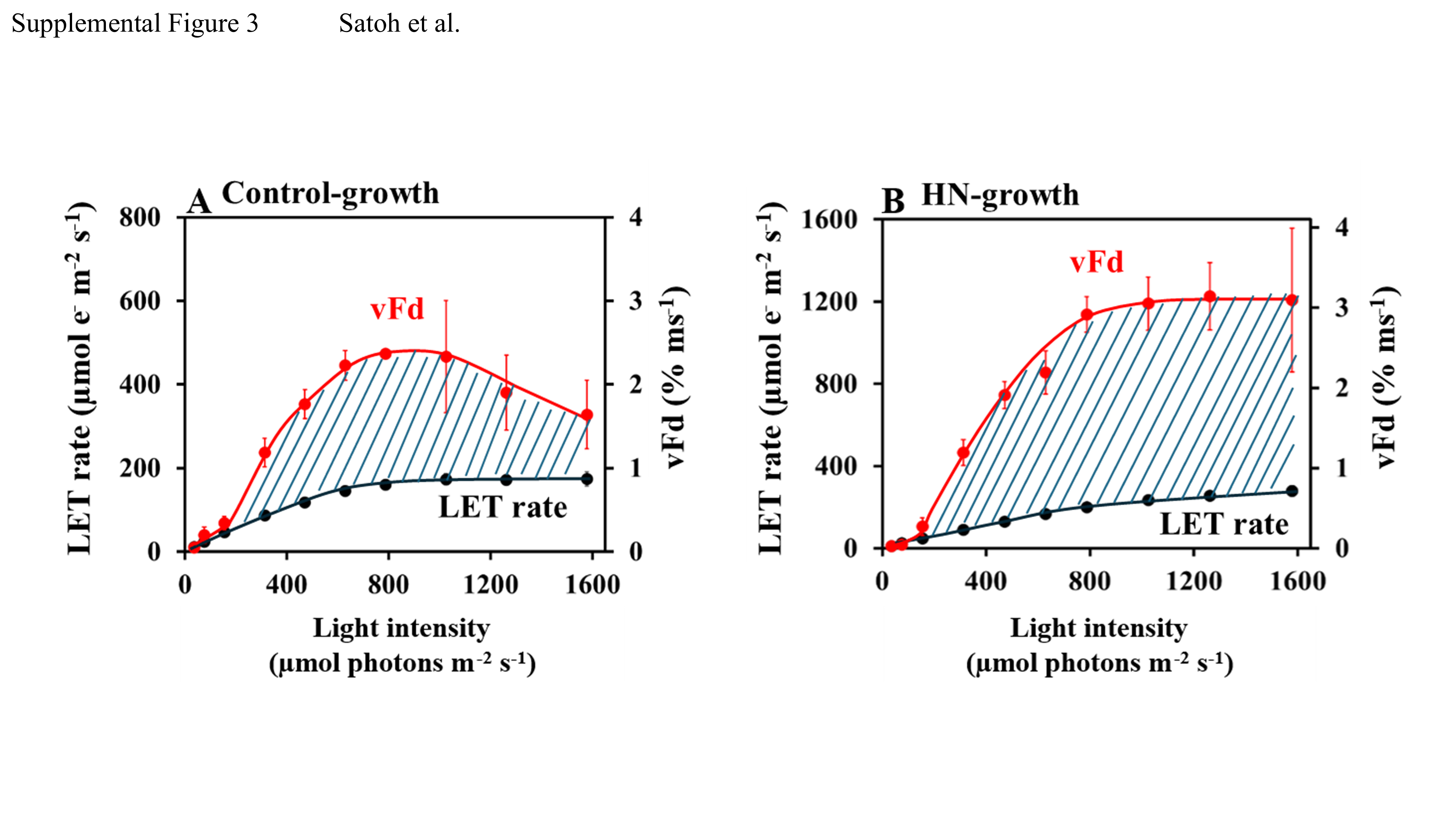

Supplement: Supplementary Figure 1 — Responses of Fd- to actinic light illumination in WT and FLV overexpressed (35S; PpFlv no.13) in Arabidopsis thaliana. Actinic light illumination (200 µmol mol photons m-2 s-1, 3 s) to the leaves of WT (A) and 35S; PpFlv no.13 (B) reduced Fd. Dark periods are indicated with a black bar, light period is indicated with a white bar. Signals of the reduced Fd were monitored by DUAL/KLAS-NIR, as described in MATERIALS AND METHODS section. [file DataSheet1.zip › Supplementary Figure S3.tif]

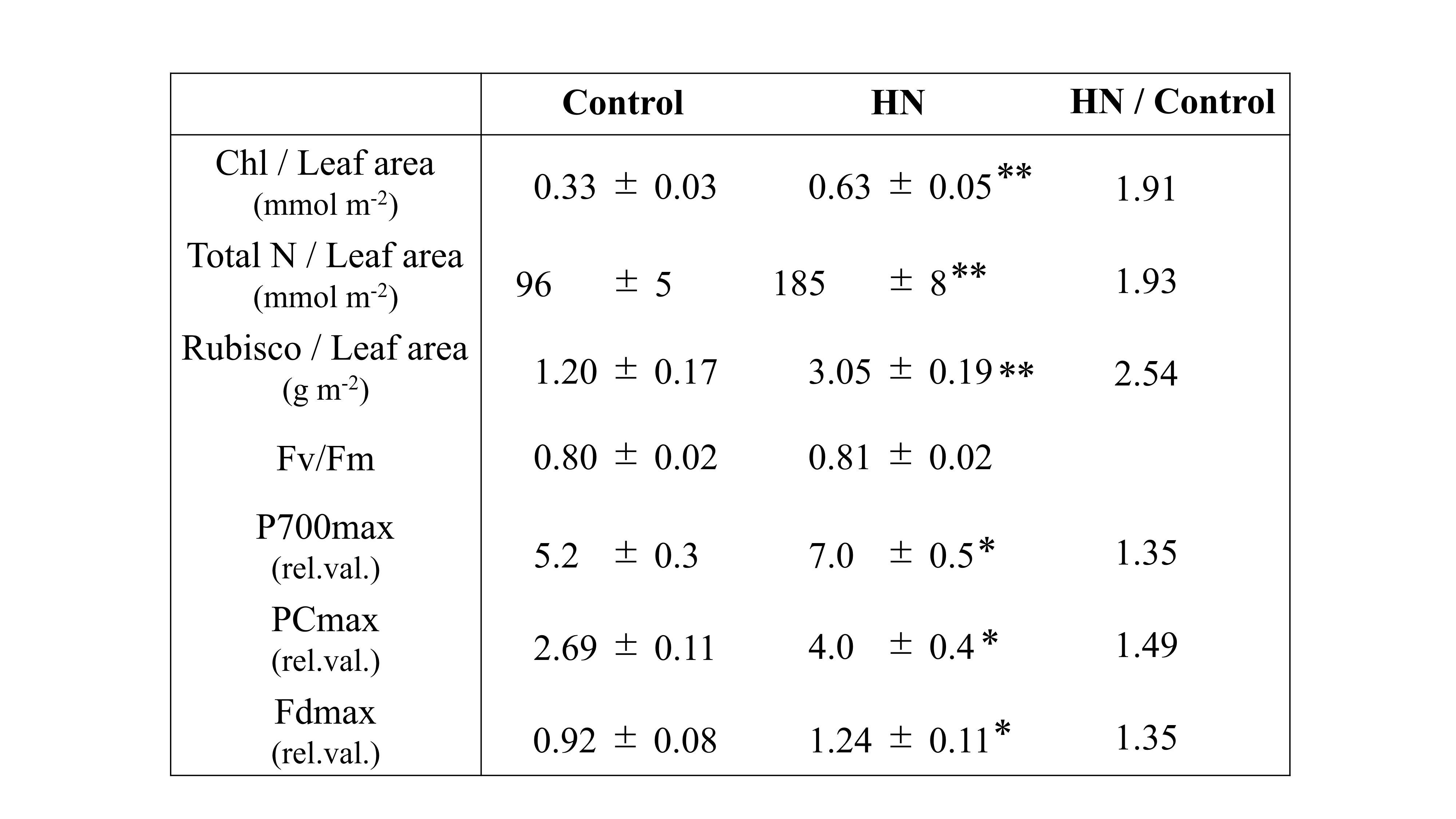

Supplement: Supplementary Figure 1 — Responses of Fd- to actinic light illumination in WT and FLV overexpressed (35S; PpFlv no.13) in Arabidopsis thaliana. Actinic light illumination (200 µmol mol photons m-2 s-1, 3 s) to the leaves of WT (A) and 35S; PpFlv no.13 (B) reduced Fd. Dark periods are indicated with a black bar, light period is indicated with a white bar. Signals of the reduced Fd were monitored by DUAL/KLAS-NIR, as described in MATERIALS AND METHODS section. [file DataSheet1.zip › Supplementary Table S1.tif]
